# Interventions to Promote Physical Activity in Older People with Type 2 Diabetes Mellitus: A Systematic Review

**DOI:** 10.3389/fpubh.2013.00071

**Published:** 2013-12-23

**Authors:** Shariff-Ghazali Sazlina, Colette Browning, Shajahan Yasin

**Affiliations:** ^1^Department of Family Medicine, Faculty of Medicine and Health Sciences, Universiti Putra Malaysia, Serdang, Malaysia; ^2^Jeffrey Cheah School of Medicine and Health Sciences, Monash University Sunway Campus, Subang Jaya, Malaysia; ^3^School of Primary Health Care, Monash University, Notting Hill, VIC, Australia

**Keywords:** physical activity, older people, type 2 diabetes mellitus, geriatric medicine, health promotion

## Abstract

**Introduction**: Type 2 diabetes mellitus (T2DM) among people aged 60 years and above is a growing public health problem. Regular physical activity is one of the key elements in the management of T2DM. Recommendations suggest that older people with T2DM will benefit from regular physical activity for better disease control and delaying complications. Despite the known benefits, many remain sedentary. Hence, this review assessed interventions for promoting physical activity in persons aged 65 years and older with T2DM.

**Methods**: A literature search was conducted using Ovid MEDLINE, PubMed, EMBASE, SPORTDiscus, and CINAHL databases to retrieve articles published between January 2000 and December 2012. Randomized controlled trials and quasi-experimental designs comparing different strategies to increase physical activity level in persons aged 65 years and older with T2DM were included. The methodological quality of studies was assessed.

**Results**: Twenty-one eligible studies were reviewed, only six studies were rated as good quality and only one study specifically targeted persons aged 65 years and older. Personalized coaching, goal setting, peer support groups, use of technology, and physical activity monitors were proven to increase the level of physical activity. Incorporation of health behavior theories and follow-up supports also were successful strategies. However, the methodological quality and type of interventions promoting physical activity of the included studies in this review varied widely across the eligible studies.

**Conclusion**: Strategies that increased level of physical activity in persons with T2DM are evident but most studies focused on middle-aged persons and there was a lack of well-designed trials. Hence, more studies of satisfactory methodological quality with interventions promoting physical activity in older people are required.

## Introduction

Type 2 diabetes mellitus (T2DM) is one of the most common chronic non-communicable diseases (NCDs) in many countries especially in the developing countries (1). The prevalence continues to increase with changing lifestyles and increasing obesity affecting all ages including older people. Current estimates indicate a growing burden of T2DM worldwide, which is greatest among persons aged 60 years and older (2, 3). Therefore, an emphasis on the lifestyle interventions such as regular physical activity to offset the trends of the increasing prevalence of T2DM is imperative. Regular physical activity is one of the key elements in the management of T2DM, and evidence has shown that engaging in regular physical activity leads to better control of T2DM and delayed complications (4, 5). Increasingly, recommendations suggest older people will benefit from regular physical activity especially in the presence of chronic NCDs such as T2DM (4, 6–8). Despite the evident health benefits, many people with T2DM, especially older people, remain sedentary or inactive (9–13).

Previous systematic reviews have been conducted to evaluate interventions promoting physical activity (14–18) but none have focused specifically on increasing levels of physical activity in people with T2DM. Only one review focused on T2DM but the review evaluated the effects of exercise on T2DM parameters and not on strategies to increase levels of physical activity (8). Only one review focused on persons aged 65 years and older, which compared the effects of home based with centre based physical activity programs on participants’ health (15). This review, however, did not include persons with T2DM. Furthermore, these reviews found that most interventions promoting physical activity had short-term effectiveness with several methodological weaknesses. To the best of our knowledge, no systematic review has been conducted evaluating interventions promoting physical activity in older people with T2DM. This review provides a qualitative evaluation of interventions promoting physical activity in older people with T2DM.

## Methods

A systematic review using a qualitative synthesis method was conducted to retrieve and review the findings of previous literature on interventions promoting physical activity in older people (aged 65 years and over) with T2DM. In this review, changes in physical activity level was selected as the outcome variable instead of changes in exercise level, as exercise is a subset of physical activity. Physical activity is defined as “body movement that is produced by the contraction of skeletal muscles and that increases energy expenditure,” while exercise is “a planned, structured, and repetitive movement to improve or maintain one or more components of physical activity” (p.1511) (6).

### Data sources and search strategy

The search was conducted electronically according to the Preferred Reporting Items for Systematic reviews and Meta-Analyses (PRISMA) guidelines (19) using the following databases: Ovid MEDLINE, PubMed, EMBASE, SPORTDiscus, and CINAHL. The Medical Subject Heading terms used in Ovid MEDLINE were adapted from Foster et al. (18) as presented in Table 1. Comparable searches were made for the other databases.

**Table 1:** **Search strategy used in Ovid MEDLINE**.

Dates 2000–December 2012	
1	Physical activity.mp
2	Exp exercise/
3	Exp walking/
4	Exp physical exertion/
5	Exp sports/
6	Exp lifestyle/
7	Exp physical fitness/
8	Strength training.mp
9	Exp resistance training/
10	Aerobics.mp
11	Physical$.mp
12	Exercis$.mp
13	Sport$.mp
14	Aerobic$.mp
15	Walk$.mp
16	Lifestyle$.mp
17 (or/1–16)
18	Exp diabetes mellitus, type 2/
19	Exp diabetes mellitus/
20 (or/18–19)
21	Exp health education/
22	Exp patient education/
23	Exp health promotion/
24	Promot$.mp
25	Educat$.mp
26	Program$.mp
27 (or/21–26)	
28 (17 and 20 and 27)	
29 [limit 28 to (English language and all aged 65 and over and RCT or quasi-experimental)]	

Only peer-reviewed published articles between years 2000 and end of December 2012 were used. No published reviews articles on physical activity were included but were used as a source of randomized controlled trials (RCTs). The reference lists of review articles and included studies were hand searched for other potentially eligible studies. Only articles published in English language were considered due to limited resources for translation. No attempts were made to contact authors for additional information, but cross-referencing on related previously published studies was performed to obtain additional information. All the titles, abstracts, and full-text of every study retrieved from the search were initially screened by one reviewer (Shariff-Ghazali Sazlina) using a standardized form with the eligibility criteria. A second reviewer (Shajahan Yasin) assessed the retrieved study if the first reviewer was in doubt on the paper’s eligibility.

### Study selection

All RCTs and quasi-experimental designs comparing different strategies to increase physical activity level in older people with T2DM were considered in this review. Studies that included self-management of diabetes and combined lifestyle (diet and physical activity) were also included. Studies with those aged 65 years and older with T2DM and living in the community were considered for this review. Studies performed on people with type 1 diabetes mellitus and impaired glucose tolerance were excluded. However, studies reporting combined results for T2DM and impaired glucose tolerance were included if the analysis of these results are conducted separately. The interventions may include one or combination of: (1) one-to-one or group counseling or advice, (2) self-directed or prescribed physical activity, (3) supervised or unsupervised physical activity, (4) on-going face to face support, (5) telephone support, (6) written motivation support material, and (7) self-monitoring devices (pedometer/accelerometer).

Interventions conducted by one or combinations of providers (health care providers, exercise specialist, peer coaches/mentors, and/or community health worker) were considered. No restrictions were included on the type and contents of the control group. The interventions could be compared with no intervention control, attention control (receiving attention such as usual diabetes care matched to length of intervention) or minimal intervention control group. The primary outcome measures in the included studies were changes in physical activity level. Studies with changes in cardiovascular disease risk factors (blood pressure, anthropometric measurements) and biochemical markers (glycosylated hemoglobin, lipid profiles) related to T2DM also were included.

### Data extraction

The data and outcomes extracted from the included studies were not combined and re-analyzed due to the qualitative nature of this systematic review and the variability in the interventions used. Each full-text article retrieved was evaluated systematically and summarized according to previously suggested method (20). These included the study’s: (1) objective (on effectiveness of physical activity interventions), (2) targeted health behavior (physical activity, self-management, or combined physical activity and nutrition), (3) characteristics of the study (study design, participants’ age, behavioral theoretical model, and sample size), (4) contents of the intervention (intervention strategies, intervention provider, length of intervention, and follow-up contacts), (5) targeted outcome(s), and (6) major results.

### Methodological quality assessment

Each of the included studies was further evaluated for its methodological quality using a list of 13 criteria adopted from an internet-based physical activity interventions systematic review (16) (see Table 2), which was based on the Cochrane Collaboration Back Review Group guidelines (21). The score to indicate good methodological quality was adopted from van den Berg et al. as there is no existing guideline on the cut-offs to rate methodological quality (16). All criteria were scored as “yes,” “no,” or “unclear” and resulting in a summary score between 0 and 13. A good methodological quality of study is considered if two thirds or more of the criteria are fulfilled, which is a summary score of 9 or higher (16).

**Table 2:** **Criteria of methodological quality**.

1	Were the eligibility criteria specified?
2	Was the method of randomization described?
3	Was the random allocation concealed? (i.e., Was the assignment generated by an independent person not responsible for determining the eligibility of the patients?)
4	Were the groups similar at baseline regarding important prognostic indicators?
5	Were both the index and the control interventions explicitly described?
6	Was the compliance or adherence with the interventions described?
7	Was the outcome assessor blinded to the interventions?
8	Was the dropout rate described and were the characteristics of the dropouts compared with the completers of the study?
9	Was a long-term follow-up measurement performed (outcomes measured ≥6 months after randomization)?
10	Was the timing of the outcome measurements in both groups comparable?
11	Was the sample size for each group described by means of a power calculation?
12	Did the analysis include an intention-to-treat analysis?
13	Were point estimates and measures of variability presented for the primary outcome measures?

*Adapted from: van den Berg et al. (16)*.

## Results

**Figure 1:**
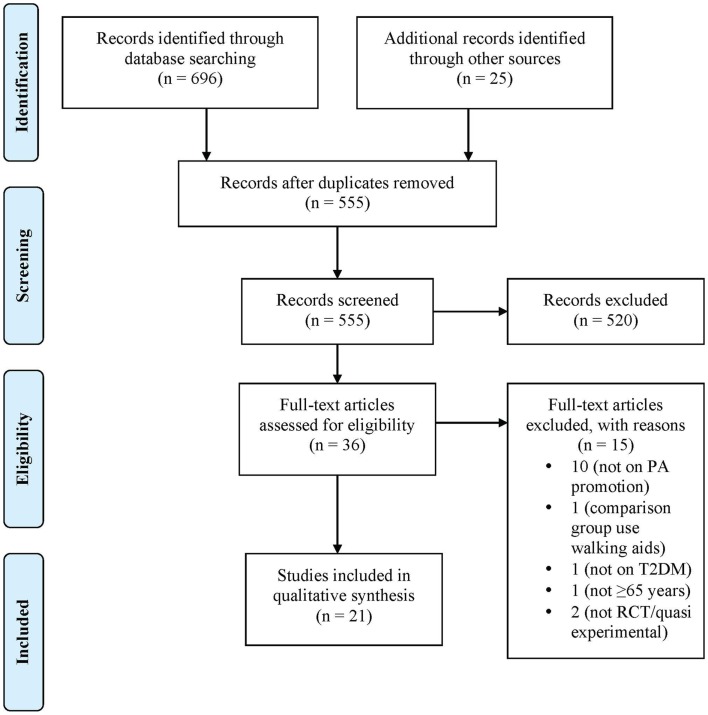
**Flow diagram for study selection according to PRISMA (19)**.

The initial search identified 696 potential articles from the database searches and another 26 were found through cross-referencing. A total of 520 studies were excluded because they did not examine physical activity, did not employ an RCT or quasi-experimental design, or did not examine T2DM or measure outcomes related to level of physical activity. A total of 36 full-text articles were selected and 21 were included in the final qualitative synthesis. Figure 1 describes the flow diagram for the study selection. We initially filtered for articles with persons aged 65 years and older, but the articles obtained from the database searches captured persons in younger age groups with some included persons aged 65 years and older. Hence, the selected studies in this review included studies that recruited both younger participants and participants aged 65 years and older.

Table 3 describes the characteristics of included studies. Eighteen studies were RCTs (22–39) and three were quasi-experimental designs (40–42). Ten studies were conducted in North America (23, 25, 27, 32, 33, 35, 37, 39–41), nine studies conducted in Europe (22, 24, 26, 28–31, 34, 38), and two studies in Australia (36, 42). About half of the included studies’ interventions focused on physical activity (22, 24, 26, 28–34, 36, 38, 41) while others on self-management of T2DM. All studies included participants aged ≥65 years with T2DM and only one study specifically studied people aged 65–80 years (23).

**Table 3:** **Characteristics of selected studies**.

Study	Methods	Quality of methods	Participants	Intervention/control or comparison group	Intervention/follow-up period and intervention provider(s)	PA/other outcomes	Summary of key findings	Notes
De Greef et al. (22)	3 Arm RCT Focus on PA Social cognitive theory	6	Primary care clinic, Belgium *N* = 67 (IG1: 22, IG2: 21, CG: 24) Aged ≤80 years, overweight (25–35 kg/m^2^) with T2DM, HbA1c ≤12%	IG1: 3 Individual counseling with goal setting by GP IG2: 3 cognitive behavioral group sessions with goal setting by psychologist CG: usual diabetes care	12 weeks/- and GP vs. psychologist	Pedometer (steps/day) IPAQ (min/day)/Weight, BMI, WC, cholesterol, FBG, HbA1c	Retention rate: 95.5% IG 2 increased steps/day (+837 ±688) than IG 1 and (+313 ±493) CG (*P* < 0.05) and total PA and MVPA min/day (*p* < 0.05) than IG1 and CG; IG1 improved WC (−1.4 cm), HbA1c (−0.32%) and total cholesterol (+7.2 mg/dl) than IG2 and CG (*p* < 0.05)	Significant findings for level of PA, HbA1c, WC, and total cholesterol
Weinstock et al. (23)	RCT Focus on self-management	8	Primary care clinic, USA *N* = 1650 (IG: 837, CG: 813) Aged 65–80 years with T2DM	IG: individual home video-conference every 4–6 weeks CG: usual diabetes care	5 years/- and diabetes educator, primary care providers	Diabetes Self-Care Activities for assessment of PA/BMI, BP, HbA1c, ADL, self-care activities, social support	Retention rate: IG had lower rate of decline in PA (*p* = 0.013) and higher self-care activity level (*p* = 0.003) than CG	Significant findings for level of PA but not for other outcomes
De Greef et al. (24)	RCT Focus on PA Social cognitive theory, motivational interviewing	8	Tertiary care clinic, Belgium *N* = 92 (IG: 60, CG: 32) Aged 35–75 years, overweight (25–35 kg/m^2^), with T2DM ≥6 months, HbA1c ≤12%	IG: 7 individual cognitive behavioral sessions (goal setting, self-efficacy, social support) and telephone support CG: usual diabetes care	24 weeks/1 year and psychologist	Pedometer (steps/day), accelerometer (min/day), IPAQ (min/day)/-	Retention rate: 95.7% at week 24: IG improved (+2744 steps/day, *p* < 0.001), total PA (+23 min/day, *p* < 0.001) and sedentary behavior (−23 min/day, *p* < 0.05) at 1 year: (+1872 steps/day, *p* < 0.001), total PA (+11 min/day, *p* < 0.001) and sedentary behavior (−12 min/day, *p* < 0.001)	Significant group difference for level of PA post intervention and at 1 year
Toobert et al. (25)	RCT Focus on self-management Social cognitive theory, goal systems	10	Primary care clinic, USA *N* = 280 (IG: 142, CG: 138) Aged 30–75 years, Latina ethnicity, T2DM ≥6 months	IG: 6×group counseling, then every 2 weeks with lay group leaders CG: usual diabetes care	1 year/- and dietitian, exercise physiologist, stress management instructor and lay group leaders	IPAQ (days/week)/BMI, BP, HbA1c, lipids, stress management, self-care, nutrition	Retention rate: 78% at 6 months IG improved in days/week exercised (*p* < 0.05), calories from fat (*p* < 0.01), and HbA1c (*p* < 0.01) than CG	Significant group difference for level of PA, fat intake and HbA1c
Wisse et al. (26)	RCT Focus on PA	7	Tertiary care clinic, Netherlands *N* = 74 (IG: 38, CG: 36)	IG: 2 personalized sessions and 2 telephone calls, and individual consultation alternate with telephone calls every 6 weeks CG: usual diabetes care	2 years/- and physio-therapist and physicians	Tecumseh/Minnesota scale: leisure time activities (MET/week)/Quality of life, BP, weight, HbA1c, FBG, lipids	Retention rate: 82.4% leisure time activities increased for IG (33 ± 4 MET/week from 15 ± 3 MET/week) and CG (39 ± 6 MET/week from 23 ± 5 MET/week) (*p* = 0.171)	No significant findings for level of PA or other outcomes
			Adults (age not stated) with T2DM, on insulin and inactive (exercise ≤160 min/week)		
Osborn et al. (27)	RCT Focus on self-management Information-motivation-behavioral skills model	6	Primary care clinic, USA *N* = 118 (IG: 59, CG: 59) Aged ≥18 years, Puerto Ricans, with T2DM >1 year	IG: group diabetes self-care counseling CG: usual diabetes care	12 weeks/- and medical assistants, dietitian, diabetes educator, psychologist	PA subscale of summary of diabetes self-care activities (SDSCA) (frequency of PA/7 days)/diet subscale of SDSCA, HbA1c, BMI	Retention rate: 77.1%. No group difference on PA scores (*p* = 0.230) and HbA1c (*p* = 0.760)? BMI results	No significant findings for level of PA or other outcomes
De Greef et al. (28)	RCT Focus on PA Motivational interviewing, cognitive behavioral	11	Tertiary care clinic, Belgium *N* = 41 (IG: 20, CG: 21) Aged 35–75 years, with T2DM ≥6 months	IG: 5 cognitive behavioral group sessions (social support, self-monitoring) and a booster session CG: usual diabetes care and one single group PA education	12 weeks/1 year and exercise coaches, clinical psychologist	Pedometer (steps/day), accelerometer (min/day)/weight, BMI, HbA1c, BP	Retention rate: 90.3% at 12 weeks, 87.8% at 1 year IG improved steps/day (*p* < 0.05) and sedentary behavior (*p* < 0.05) post intervention than CG, not at 1 year	Significant group difference on PA level only at post intervention
Balducci et al. (29)	RCT Focus on PA	10	Tertiary care clinic, Italy *N* = 606 (IG: 303, CG: 303) Aged 40–75 years, with T2DM and sedentary (? definition)	IG: 2 supervised exercise sessions/week, 4 individual exercise counseling CG: usual diabetes care and exercise counseling	1 year/- and exercise specialist and diabetologist	Minnesota Leisure time PA questionnaire (MET h/week)/HbA1c, lipids, BP, indirect VO_2max_, flexibility	Retention rate: 92.9% IG improved in MET h/week (mean diff. +10.00, *p* < 0.001), VO_2max_ (2.8, *p* < 0.001), HbA1c (−0.30%, *p* < 0.001), systolic BP (−4.2 mmHg, *p* = 0.002), diastolic BP (−1.7 mmHg, *p* = 0.030) HDL-C (+3.7 mg/dl, *p* < 0.001), and LDL-C (−9.6 mg/dl, *p* = 0.003); WC (−3.6 cm, *p* < 0.001) than CG	Significant group difference on PA level, VO_2max_, HbA1c, BP, HDL-C, LDL-C, and WC
Negri et al. (30)	RCT Focus on PA	7	Tertiary care clinic, Italy *N* = 59 (IG: 39, CG: 21) Aged 50–75 years, inactive (? definition), T2DM ≥2 years, HbA1c 6.5–9.9%	IG: 3 supervised walking group/week, one individual and one group counseling CG: standard lifestyle advice	16 weeks/- and personal exercise trainer	Activity log (MET h/week)/HbA1c, FBG, lipids, 6 min walk test	Retention rate: 86.4% IG improved MET h/week (*p* = 0.008), HbA1c (*p* = 0.01), and distance walked in 6 min (*p* = 0.001) than CG	Significant group difference on PA level, HbA1c and 6 min walk test
Kirk et al. (31)	3 arm RCT Focus on PA Trans theoretical model	11	Multifaceted care, UK *N* = 134 (IG1: 47, IG2: 52, CG: 35) Inactive (? definition) adults (age not stated) with T2DM	IG1: written self instructional walking plan (with goal setting) IG2: written self instructional walking plan (with goal setting) with 2 individual consultation CG: usual diabetes care and a leaflet on PA	1 year/- and research team	Accelerometer (h/day), 7-day recall questionnaire/HbA1c, BMI, WC, BP, lipids	Retention rate: 86.6% No group difference on accelerometer (*p* = 0.863), step counts (*p* = 0.739), minutes of moderate PA/week (*p* = 0.212). Time effects on HbA1c (*p* = 0.026), total cholesterol (*p* = 0.001), HDL-C (*p* = 0.029), WC (*p* = 0.020), systolic BP (*p* = 0.037), and diastolic BP (*p* = 0.001)	No group difference PA level or other outcomes, significant time effects on HbA1c, lipid profiles, BP, and WC
Dutton et al. (32)	RCT Focus on PA Trans theoretical model, social cognitive theory	7	Primary care clinic, USA *N* = 85 (CG: 39; IG: 46) Aged ≥18 years with T2DM	IG: one-to-one tailored print-based PA counseling motivation (included self-efficacy, goal setting, social support) CG: diabetes specific dietary tip sheet advice, no advice on PA	4 weeks/- and research team	7-day PA recall for MVPA (min/week)/-	Retention rate: 94.0%. No group difference on min/week of PA (*p* = 0.220)	No group difference on level of PA
Allen et al. (33)	Pilot RCT Focus on PA Self-efficacy theory	7	Primary care clinic, USA *N* = 52 (CG: 25; IG: 27) Aged >18 years with T2DM, not on insulin, inactive (<3 days/week of physical activity), HbA1c >7.5%	IG: individual glucose monitoring counseling, feedback from glucose chart and one telephone call (goal setting, problem solving) CG: individual diabetes education and one telephone call	8 weeks/- and research team	Accelerometer (min/day)/BP, BMI HbA1c, Self-efficacy for exercise behavior	Retention rate: 88.5% IG improved light/sedentary activity (−2.7 ± 4.8 min/day, *p* < 0.05), moderate activity (5.5 ± 2.9 min/day, *p* < 0.05), HbA1c (−1.2 ± 1.0%, *p* < 0.05), and BMI (0.5 ± 0.7 kg/m^2^, *p* < 0.05) than CG	Significant group difference on PA level, HbA1c, and BMI
Bjørgaas et al. (34)	RCT Focus on PA	7	Tertiary care clinic, Norway *N* = 69 (IG: 31, CG: 37) Aged <80 years with T2DM	IG: 2 individual PA sessions + pedometer use (self-monitoring) CG: 2 individual PA sessions	24 weeks/- and Research team	Questionnaire on physical fitness and activity, exercise testing using VO_2peak_ (l/min)/HbA1c, FBG, lipids	No group difference on the physical fitness and activity scores (*p* > 0.800), health outcomes (*p* > 0.640), VO_2peak_ (*p* > 0.170). CG increased VO_2peak_ over time (*p* = 0.036)	No group difference on PA levelor other outcomes; CG had increased VO_2peak_ over time
Toobert et al. (35)	RCT Focus on self-management Social cognitive theory, goal systems, social ecological theory	11	Primary care clinic, USA *N* = 279 (IG: 163, CG: 116) Aged <75 years, post menopausal women, T2DM ≥6 months	IG: 6×group counseling and support CG: usual diabetes care	1 year/1 year and dietitian, exercise physiologist, stress management instructor, lay group leaders	CHAMPS (kcal/kg/h of moderate intensity PA)/diet, flexibility, stress management, social support, problem solving, self-efficacy, depression, quality of life	Retention rate: 85.0% IG improved kcal/kg/h of moderate intensity PA (*p* < 0.01), min/day of stress management practice (*p* < 0.001), calories of saturated fat (*p* < 0.001) and sit-reach % score (*p* < 0.05) than CG	Significant group difference on PA level, saturated fat intake, stress management and flexibility
Engel and Lindner (36)	RCT Focus on PA	6	Community, Australia *N* = 57 (CG: 30; IG: 24) Aged 50–70 years with T2DM, sedentary (≤30 min/week of physical activity)	IG: 6 individual health related coaching + pedometer use (feedback, self-efficacy, goal setting) CG: 6 individual health related coaching	24 weeks/- and research team	Activity log (min/day of walking activity)/HbA1c, weight, BMI, BP, shuttle test (cardio respiratory fitness)	Retention rate: 88.0% no group difference on time spent walking (*p* = 0.207) and other outcomes. Significant time effects on PA (*p* < 0.001), weight (*p* < 0.05), WC (*p* < 0.001), and shuttle test (*p* < 0.001)	No group difference on PA level or other outcomes; Significant time effects over time for PA, weight, WC, and cardio respiratory fitness
King et al. (37)	RCT Focus on self-management Goal system theory, social cognitive theory, social ecological theories	6	Primary care clinic, USA *N* = 335 (IG: 174, CG: 161) Aged ≥25 years, T2DM ≥6 months	IG: individual self-management counseling (interactive CD-ROM) with goal setting, 2 follow-up telephone calls and a tailored health newsletter CG: one visit at enrolment for an interactive computerized health risk appraisal and brief health counseling	8 weeks/- and Health coaches	CHAMPS questionnaire (kcal/kg/h and total caloric expenditure/week)/dietary pattern	Retention rate: 92.2% IG improved all PA (*p* < 0.01), moderate PA (=0.001), and strength training (*p* < 0.001) than CG	Significant group difference on level of PA
Kirk et al. (38)	RCT Focus on PA Trans theoretical model, motivational theory, cognitive behavioral strategies	8	? Setting, UK *N* = 70 (IG: 35, CG: 35) Adults (age not stated) with T2DM	IG: one individual exercise consultation with exercise leaflet and 2 follow-up telephone calls (goal setting, social support) CG: exercise leaflet (part of usual diabetes care) and 2 follow-up telephone calls	24 weeks/- and research team	7-day PA recall (min/week), accelerometer (activity counts/week)/indirect VO_2max_, stage, and processes of change, BP, BMI, HbA1c, lipids, fibrinogen	Retention rate: 90.0% IG improved moderate activity PA (*p* < 0.001), activity count/week (*p* < 0.001), total exercise duration, and peak gradient (*p* < 0.005), HbA1c (*p* = 0.02) and systolic BP (*p* = 0.02) compared with CG	Significant group difference on PA level, HbA1c, and systolic BP
Keyserling et al. (39)	3 arm RCT Focus on self-management Behavior change theory	10	Primary care clinic, USA *N* = 200 (IG1: 67, IG2: 66, CG: 67) Aged ≥40 years African American women with T2DM	4 Individual clinic based counseling alone (IG1) or combined with 3 group sessions and 12 telephone calls (IG2) CG: received mailed pamphlet on PA, nutrition, and diabetes	1 year/- and primary care physicians, community diabetes advisor, peer counselors	Accelerometer (kcal/day)/dietary intake, HbA1c, lipids	Retention rate: 85.5% IG2 (44.1 kcal/day, *p* = 0.006) and IG1 (33.1 kcal/day, *p* = 0.029) had higher mean kcal/day than CG. No group difference on the other outcomes	Significant group difference on PA level, not for other outcomes and dietary intake
Diedrich et al. (40)	Quasi-experimental Focus on self-management Social cognitive theory	6	Tertiary care clinic, USA *N* = 53 (IG: 27, CG: 26) Aged 23–89 years with T2DM	IG: diabetes self-management education (DSME) programs + pedometer use (goal setting, self-monitoring) CG: DSME	12 weeks/- and diabetes nurse and dietitian	Paffenbarger PA questionnaire (total scores)/HbA1c, BP, BMI, body fat	Retention rate: 62.0% IG improved diastolic BP (*p* = 0.024) than CG; Effect of time: IG improved in HbA1c (*p* = 0.020) and body fat (*p* = 0.037); CG improved in HbA1c (*p* = 0.005) and weight (*p* < 0.001)	Significant group difference on diastolic BP but not for PA. Significant time effect on HbA1c, body fat, and weight
Tudor-Locke et al. (41)	Quasi-experimental Focus on PA Social cognitive theory	7	Tertiary care clinic, Canada *N* = 220 (CG: 157; IG: 63) Aged 40–70 years with T2DM, inactive (walks <8800 steps/day)	4 Group sessions followed and 12 self-directed behavior change (goal setting, self-monitoring and feedback) by healthcare professionals (IG) or by peers (CG)	16 weeks/- and Health care professionals vs. peers	Pedometer (steps/day)/Weight, WC, resting HR, BP	Retention rate: 75.0%. No group difference on all outcomes; Effect of time: both IG and CG improved steps/day (*p* < 0.001), weight (*p* < 0.001), WC (*p* < 0.001), and BP (*p* < 0.001)	No group difference on PA level or other outcomes; Significant time effects on PA, weight, WC, and BP
Furber et al. (42)	Quasi-experimental Focus on self-management Social cognitive theory	6	Community, Australia *N* = 226 (IG: 121, CG: 105) Adults (age not stated) with T2DM or impaired glucose tolerance	IG: one group education session + pedometer use (goal setting, self-monitoring) Length: 2 weeks, follow-up at 20 weeks	2 weeks/20 weeks and diabetes nurse educator, dietitian CG: one group education session	Active Australia survey on PA (min/week)/-	Retention rate: 92.9% at week 2; 81.4% at week 20 IG improved time spent walking (mean diff. 59.4 min/week, *p* < 0.05) and moderate intensity PA (*p* < 0.05) post intervention than CG; No group difference at week 20	Significant group difference on PA level

*PA, physical activity; IG, intervention group; CG, control or comparison group; GP, general practitioner; IPAQ, international physical activity questionnaire; MET, metabolic equivalent time; CHAMPS, community healthy activities program for seniors; BMI, body mass index; WC, waist circumference; FBG, fasting blood glucose; HbA1c, glycosylated hemoglobin; BP, blood pressure; LDL-C, low density lipoprotein cholesterol; HDL-C, high density lipoprotein cholesterol; ADL, activities of daily livings; MVPA, moderate-to-vigorous physical activity*.

*A summary score of 9 or higher indicate good methodological quality*.

The type of interventions used in each study varies markedly as shown in Table 3. Most interventions were delivered either as a group (22, 24, 25, 27, 28, 30, 35, 39, 41, 42) or using one-to-one counseling/advice (23, 24, 26, 29, 31–34, 36–38, 40). The majority of the studies’ interventions were delivered by one or more healthcare providers (22–30, 35, 37, 39–42) and some included peers as the interventionists (25, 35, 39, 41). In order to provide support and motivation, seven studies contacted the participants on ≥2 occasions in the first 4 weeks of the intervention (24–26, 29, 30, 35, 37).

Most studies incorporated one or a combination of health behavior theories in their interventions and social cognitive theory was the most commonly adopted theory (22, 24, 25, 32, 37, 40–42). Half of the included studies’ interventions were compared with control groups receiving usual diabetes care alone (22–27, 35). The outcome measures and results of interventions promoting physical activity are presented in Table 2. In most studies the primary outcome was either level of physical activity alone, or physical activity level in combination with other health outcomes. The level of physical activity were measured objectively using pedometer and/or accelerometer (22, 24, 28, 31, 33, 38, 39, 41) in combination with a questionnaire (22, 24, 31, 38). Eleven studies assessed level of physical activity subjectively using only a questionnaire (23, 25–27, 29, 32, 35–37, 40, 42), the content of which varied widely. The unit of measurement to represent the level of physical activity also varied.

Ten of the 12 studies which compared the physical activity intervention to a control group reported a significant increase in the level of physical activity in the intervention group (22–25, 28–30, 35, 37, 39). Some studies also reported improvements in HbA1c level (22, 25, 29, 30), other CVD risk factors (blood pressure, waist circumference, and lipid profiles) (22, 29) and in cardiorespiratory fitness (30). Nine studies which did not differ in number of contacts, but only on treatment procedure between the intervention and comparison groups, showed no difference between groups on physical activity level and CVD risk factors (31, 32, 34, 36, 41). Six of the 21 studies fulfilled nine or more criteria of methodological quality implying good quality studies (see Table 3) (25, 28, 29, 31, 35, 39). Only three studies applied intention-to-treat analysis principles (25, 30, 31). Studies with lower scores demonstrated methodological weaknesses related to randomization processes, sample size estimation, and outcomes assessment processes.

## Discussion

This review identified 21 studies (18 RCTs and 3 quasi-experimental designs) that promoted physical activity in persons with T2DM, which involved older people. These studies were conducted in eight countries with none from the Asian region. The majority of the studies had participants in the middle age groups and only one study specifically recruited participants aged ≥65 years. Half of the studies focused on physical activity, while others focused on the self-management of diabetes. From this review, it is evident that significant heterogeneity in the interventions existed making comparisons difficult and any general conclusions must be made with caution.

The levels of physical activity of the participants often differed at randomization; hence, it was difficult to make valid conclusions about the effectiveness of these interventions. From this review, only three studies controlled for baseline physical activity. Other studies either controlled for variables that differed at baseline or there was no difference between groups at baseline and therefore the authors did not report controlling for baseline physical activity (27, 29, 32). Only a third of the studies targeted sedentary or inactive participants at recruitment, but the definition of sedentary or inactivity varied greatly (26, 29–31, 33, 36, 41). In some studies, the participants were asked to build on their present physical activity; hence, these participants may be physically active at recruitment. Participants who are already physically active are more likely to comply with physical activity interventions and maintain a healthy lifestyle than those who are sedentary or inactive (43).

Both one-to-one and group sessions improved the level of physical activity. However, most of these studies incorporated multiple constructs from health behavior theories including strategies such as goal setting, problem solving, self-monitoring, and social support in their interventions. It is assumed that these approaches incorporate multiple constructs and strategies to facilitate behavior change and maintenance (44). The constructs of social cognitive theory such as self-efficacy and social support were the most frequently used, with positive results in changing physical activity level (22, 24, 25, 33, 35, 37, 42) and improving glycemic control (22, 25, 33). However, this review is not able to provide the evidence to recommend the most suitable health behavior theories for future interventions. Some studies incorporated more than one health behavior theory in their interventions making comparison between studies difficult.

Interventions promoting physical activity with follow-up contacts during the study period did increase the level of physical activity and improved control of glycemia and other CVD risk factors. Five studies had a long period of intervention of at least 1-year duration (23, 25, 29, 35, 39) with reported long-term effects of the interventions for the level of physical activity. The effects of follow-up contacts with the intervention provider and long intervention duration could influence the observed positive outcomes in these studies.

The majority of the studies measured the level of physical activity as the primary outcome and most studies used a single physical activity outcome measure, predominantly validated self-reported scales or an activity log (23, 25–27, 29, 30, 32, 35, 36, 40, 42). Most of these studies did *not use objective measures to assess* the change in the level of physical activity but use self-report measures to obtain energy expenditure, total scale scores, oxygen uptake or the relative change in duration, frequency, and/or intensity of physical activity. Some studies did use objective measures such as motion sensor devices (accelerometer and/or pedometer) (22, 24, 28, 31, 33, 38, 41). However, self-reported physical activity scales do lack validity in measuring physical activity and were found to be inferior to the motion sensor devices (45, 46). This would lead to less precise measurement and misclassification of the level of physical activity. Hence, an objective measure of physical activity is necessary to establish the effect of intervention in a trial, as it allows a uniform measurement of the physical activity level.

In this current review, healthcare providers delivered the majority of the studies’ interventions and they may be more motivated to deliver the interventions than they might in a non-trial setting. In addition, the participants in most of these studies had to undergo extensive screening prior to randomization, and hence, participants who finally participated in these studies were more likely to be highly motivated (16). The evidence of effectiveness is also limited by the control or comparison groups, which varied widely. In some studies participants in the control group received only usual diabetes care or more general information about lifestyle changes while others received additional counseling about physical activity and some had multiple counseling sessions on diabetes self-care management. A number of studies included feedback from pedometer use, goal setting, and social support in the control/comparison groups as received by the intervention group as these studies were assessing a specific component of their intervention such as who delivers the interventions.

The methodological quality of the included studies in this review varies. Only six studies (all RCTs) were rated as good quality. The quality of the included studies in this review was limited by a lack of intention-to-treat analysis as only three studies perform such analysis. The studies with low scores have weaknesses in terms of inadequate description of the randomization methods; no information on random assignment performed by an independent person, insufficient description of sample size estimation and lack of information on whether an independent assessor assesses the main outcome measures. Inadequate methodological approaches in trials are associated with bias (47).

This review included multiple major databases with vigorous and systematic search strategy. However, there are limitations from this review. Only peer-reviewed papers published in recent years (i.e., from year 2000) and published in English are included in the data extraction, hence a possibility of selection bias exists. In addition, even though the searches are done thoroughly through multiple major databases with cross-referencing; there is a possibility that some papers are not included due to the inclusion criteria used for this current review. In this review, only one reviewer assessed the studies for eligibility, which could contribute to an increased risk of evaluation bias.

## Conclusion

The number of well-designed trials on interventions promoting physical activity in older people with T2DM is limited as evident in this present review. The methodological quality, type of interventions promoting physical activity and outcome measure for level of physical activity in the included studies included in this review differed widely. Studies with interventions promoting physical activity that compared with usual diabetes care do have significant findings in changing the level of physical activity in persons with T2DM. Moreover, on-going follow-up support seems to contribute in increasing level of physical activity. However, these studies are restricted to middle-aged persons with T2DM in western countries. In addition, very few studies had follow-up assessment post intervention to allow evaluation on sustainability of interventions promoting physical activity. Peer support for adults with T2DM may have potential in promoting physical activity but the evidence is scarce. Furthermore, standardization on the measure for physical activity with the use of objective tool such as the pedometer or the accelerometer is needed to allow a uniform classification of level of physical activity. Therefore, further exploration in these areas is warranted when developing interventions to promote physical activity in older people with T2DM.

## Authors Contribution

Colette Browning conceived the primary research question for the study. Shariff-Ghazali Sazlina, Colette Browning, and Shajahan Yasin were involved in the study conception and design. Shariff-Ghazali Sazlina was responsible for data extraction and Shajahan Yasin assessed any doubtful papers. Shariff-Ghazali Sazlina interpreted the results and drafted the initial manuscript. Colette Browning and Shajahan Yasin provided input on interpretation of results and provided critical revision to the manuscript for important intellectual content. All authors read and approved the final manuscript.

## Conflict of Interest Statement

The authors declare that the research was conducted in the absence of any commercial or financial relationships that could be construed as a potential conflict of interest.
